# Children and Adolescents Mental Health: A Systematic Review of Interaction-Based Interventions in Schools and Communities

**DOI:** 10.3389/fpsyg.2019.00918

**Published:** 2019-04-24

**Authors:** Rocío García-Carrión, Beatriz Villarejo-Carballido, Lourdes Villardón-Gallego

**Affiliations:** ^1^Faculty of Education, Psychology and Social Work, Universitat de Lleida, Lleida, Spain; ^2^Faculty of Psychology and Education, University of Deusto, Bilbao, Spain

**Keywords:** interaction-based interventions, mental health, schools, communities, children, adolescence, systematic review

## Abstract

**Background:** There is growing evidence and awareness regarding the magnitude of mental health issues across the globe, starting half of those before the age of 14 and have lifelong effects on individuals and society. Despite the multidimensional nature of this global challenge, which necessarily require comprehensive approaches, many interventions persist in seeking solutions that only tackle the individual level. The aim of this paper is to provide a systematic review of evidence for positive effects in children and adolescents' mental health resulting from interventions conducted in schools and communities in which interaction among different agents is an integral component.

**Methods:** An extensive search in electronic databases (Web of Knowledge, SCOPUS, ERIC, and PsycINFO) was conducted to identify interventions in which interactions between peers, teachers, families or other community members or professionals played a role. Their effects on children and adolescents' mental health were also reviewed. We carried out a systematic review of papers published from 2007 to 2017. Eleven studies out of 384 met the inclusion criteria. Seven of the articles reviewed focus on interventions conducted in schools and promote supportive interactions involving students, teachers, families and mental health professionals. Four of the articles develop interventions that engage community members in dialogic interactions with children and adolescents.

**Results:** Interventions in schools and communities implement strategies that foster supportive interactions among diverse actors including teachers, parents, community members, and other professionals. The effects of the mental health interventions reported on children and adolescents' problems include a decrease in disruptive behaviors and affective symptoms such as depression and anxiety, together with an increase in social skills, as well as an improvement in personal well-being.

**Conclusions:** There is evidence of a positive effect on the mental health of children and adolescents, both in decreasing symptoms of mental disorder and in promoting emotional well-being. Whereas, interactions among different actors seem to be a relevant aspect across the interventions, more research is needed to conclude its effect on the outcomes of the studies reviewed.

## Introduction

Childhood and adolescence are critical periods to promote mental health as more than half of mental health problems start at these stages, and many of these persist throughout adult life (Kessler et al., 2005). Currently, this has become a priority as worldwide data shows an increase in the prevalence of mental health issues in childhood and adolescence (de la Barra M, 2009) and the percentage of those afflicted reaching nearly 20% (WHO, 2016). The situation is further exacerbated by the fact that many of these children and adolescents are not receiving the specialized care they require (Mills et al., 2006; Weist and Murray, 2008; Green et al., 2013).

Consequently, important efforts to bring together the best evidence about mental health have been done and raised the challenge of agreeing about fundamental issues in the field such as the definition of mental health and other related concepts (Mehta et al., 2015). According to WHO, mental health is understood not as a mere absence of illness, but rather, in a broader sense, as a state of well-being in which individuals develop their abilities, face the stress of daily life, perform productive and fruitful work, and contribute to the betterment of their community (WHO, 2004). This definition served as the basis for WHO Mental Health Action Plan, 2013–2020, which incorporates the concepts of mental health promotion, mental illness prevention and treatment, and rehabilitation. Particularly, developmental aspects of children and young people, including, for instance, the ability to manage thoughts, emotions, as well as to build social relationships, and the aptitude to learn, are emphasized in the plan as critical facets to be tackled in mental health interventions.

Mental health interventions conducted in schools and in the communities start from the premise that the problems experienced by adolescents are determined by the interaction of individual, environmental and family factors (Manjula, 2015). Accordingly, schools and communities offer an optimal context to intervene as children and adolescents grow and develop through social interaction. Schools and communities can make the most of its environment to foster child and youth development and to promote good mental health (Weist and Murray, 2008). Many of the mental health programs implemented in schools promote the development of social skills, socio-emotional competences, and learning outcomes while at the same time reducing disruptive behavior (Dowdy et al., 2010; Moreira et al., 2010; Durlak et al., 2011; Suldo et al., 2014). The school environment and climate can therefore play a critical role in encouraging the promotion of protective factors for mental health, such as social-emotional competences and skills (Osher et al., 2012).

Hence, social and cognitive development is enacted through social interactions in a particular cultural and social context (Vygotsky, 1978; Bronfenbrenner, 1979). Drawing on the contributions of Vygotsky's theory of cognitive development, human interaction that takes place in the social and cultural context enhances learning and is fundamental for psychological function. These cultural processes in which people learn and developed occur through interactions with others, including symmetrical (peer) as well as expert–novice (e.g., teacher–student) relations (Rogoff, 1990; Cole, 1996). Importantly, specific instruments have been produced to capture productive forms of dialogue across educational contexts (Hennessy et al., 2016).

Most of the research have been devoted to understanding the central role played by the quality of dialogue and interaction between students, in small group classrooms, or in whole class setting teacher-student interaction (see review by Howe and Abedin, 2013). Furthermore, research conducted in community-based schools has also reported the benefit of involving families and community members in learning interactions with elementary students, especially for those belonging to vulnerable populations (Flecha and Soler, 2013; Valls and Kyriakides, 2013). Accordingly, community plays a central role as human develop through their interactions in the sociocultural activities of their communities (Rogoff, 2003). Similar improvements have been reported among students with disabilities as a result of engaging in caring and supportive interactions among peers and with other adults when solving academic tasks in interactive groups (García-Carrión et al., 2018). The relevance of productive forms of dialogue and supportive interactions among peers, teachers and other community members, have also reported positive effects in 4th grade students prosocial behavior (Villardón-Gallego et al., 2018). These studies evidence the potential of educational interventions that draw on the potential of fostering interactions among different agents and promote productive dialogues as a tools for academic and social improvement.

However, when searching for mental health improvement through dialogic interactions, the research is scarce. The pioneering study carried out by Seikkula and Arnkil (2006) showed the psychological and social benefits of the therapy based on open and anticipation dialogues with adults and adolescents that also involved the family along with the professionals. Rather than focusing in the individual, facilitating supportive interactions among peers, professionals and family members might be an asset underpinning mental health interventions with children and adolescents. This study showed the critical role of collective interactions, which were very different from a dialogue between two individuals (Seikkula and Arnkil, 2006). They identified multi-system treatments (MST) characterized by engaging in close interaction professionals with adolescents, family, and other networks. Replication of these US studies in Norway found evidence of effectiveness, particularly, in the adolescents' social skills (Ogden and Halliday-Boykins, 2004). However, according to Seikkula and Arnkil (2006, p. 181): “what ultimately caused the observed outcome was not revealed. After all, methods do not help or cure anyone as such. Psychological methods -and other interaction-based means- exist as they user activity.”

Whereas, determining the effect of the interaction itself in the outcomes obtained might be problematic, the authors of these paper aim to examine interaction-based mental health interventions, defined as those in which collective interactions, that involve professionals, family and community members with children and adolescents, are an integral component of the intervention. This systematic review focuses on those interventions conducted in schools and communities and its outcomes on children and adolescents' mental health. According to the WHO definition of mental health provided above, primary studies selected for this review will include positive outcomes in a broader sense, comprising not only the reduction of symptoms of mental disorder but also the promotion of emotional well-being.

## Methods

The study carries out a systematic review (Gough et al., 2013), a methodology developed by the EPPI Centre of the UCL Institute of Education. We have also taken into account the recommendations by PRISMA (Moher et al., 2009) and checklist by Joanna Briggs Institute (JBI) (Lockwood et al., 2015), in order to offer transparency, validity, replicable, and updateable in this study.

### Search Strategy

This systematic review has been focused and defined by the question: Do interaction-based mental health interventions in schools and communities have positive effects among children and adolescents? This question has been defined in terms of PICOS: In children and adolescents (Population) are interaction-based interventions (Intervention) effective in decreasing disruptive behaviors and affective symptoms such as depression and anxiety (in children and adolescents with mental health problems), and in increasing social skills, and improving well-being and academic engagement (in children and adolescents in general)? (Outcomes).

For the review, empirical articles published in international scientific journals in the areas of psychology, education, and mental health and focused on interventions among children and youth between 2007 and 2017 were searched and screened. To that effect, the following databases were analyzed: Web of Knowledge, SCOPUS, ERIC, and PsycINFO.

The articles were searched using the following keywords: “school-based,” “community-based,” “dialogue,” “mental health,” “well-being,” “emotional development,” “interventions,” “program,” “interaction,” and “prevention.” The exploration was completed with searches that employed synonyms or derivatives of the keywords. The keywords were also combined to refine the search. The publications containing the search criteria in the title, in the keywords and in the abstract were include.

### Inclusion and Exclusion Criteria

In order to identify and select the studies most relevant to our research, inclusion and exclusion criteria were established.

The inclusion criteria were the following:

- Special population group: children and adolescents.
- Target age: 6 to 18 years of age, inclusive.
- Mental health interventions in which collective interactions, including professionals, families, and community members with children and adolescents, are an integral component.
- Studies reporting outcomes of the intervention in decreasing symptoms and/or promoting well-being.

The exclusion criteria were the following:

- Interventions focus on early childhood, youth, or adults.
- Target age is not specified, or the target population is below 5 or above 18 years.
- Mental health interventions focusing on one-to-one interactions (i.e., professional-child/professional-adolescent).
- The intervention is not described or assessed, as in trials, theoretical research or literature reviews.

### Selection Process

The first part of the search yielded a total of 384 articles from indexed journals: 183 in published in the WOS database, 12 in Scopus, 33 in ERIC and 156 in PsycINFO. All these articles were entered into the Mendeley software for its screening and review. Basic information such as the title, year, authoring, and abstracts was obtained and introduced in a spreadsheet for a first screening.

From the 384 articles gathered in the initial search, the titles and their authors were subsequently revised in order to eliminate duplicates. This review was carried out by the members of the group independently in order to eliminate duplicate documents, specifically 83 were duplicates and were therefore discarded, resulting in a new total of 301 articles.

**Figure 1:**
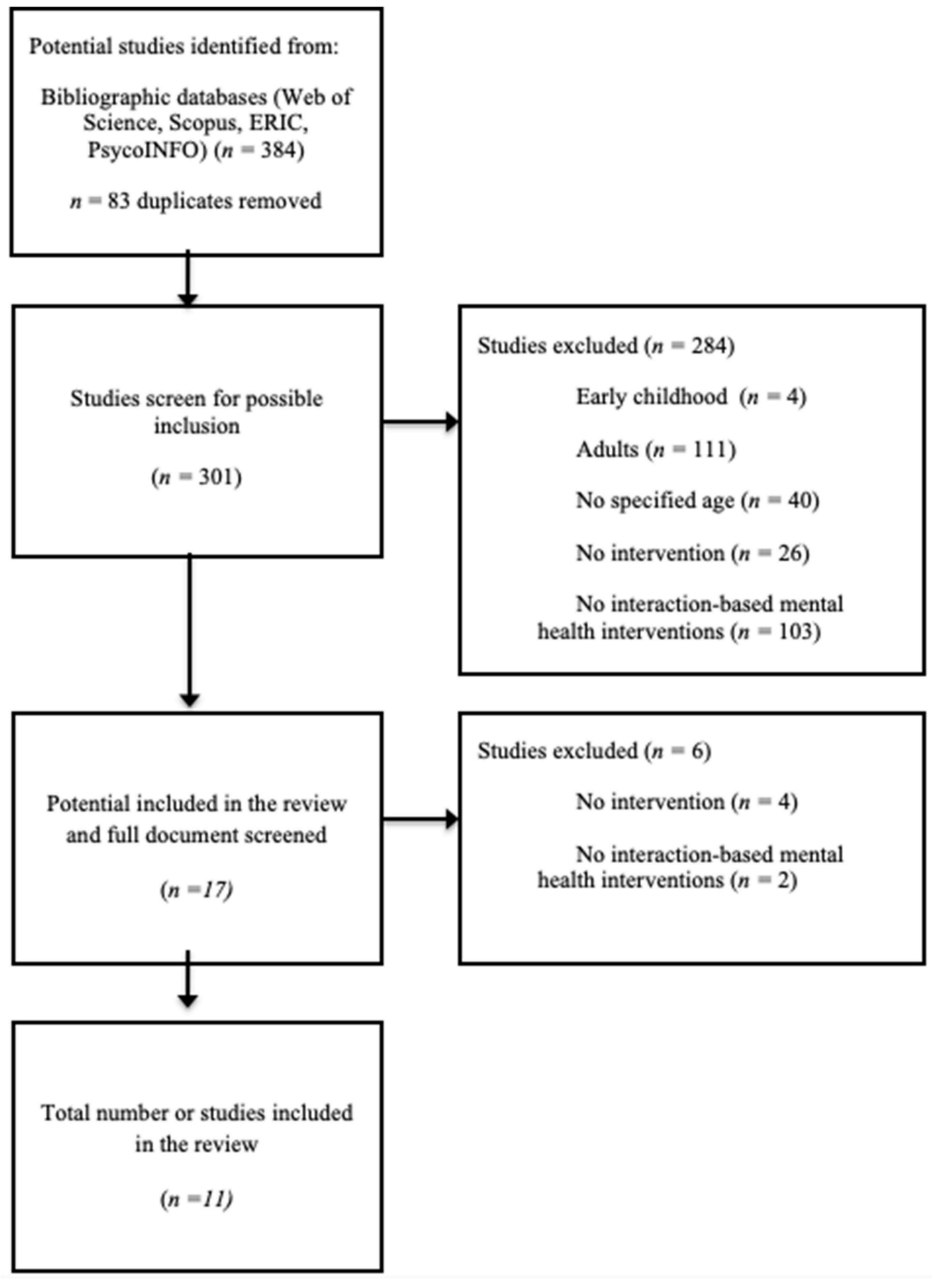
Flow diagram to show the process of study selection.

Abstracts of the 301 articles were reviewed according to the inclusion and exclusion criteria. As a result, 17 articles initially met the inclusion criteria and were eligible for the review (see Figure 1). The articles were downloaded for an in-depth review.

The three researchers examined the articles independently and extracted the most relevant information that was included in a spreadsheet. The information referred to: (a) study characteristics (author, country, selection criteria, design, data acquisition period), (b) population (target population, age and sample size), (c) settings, and (d) type of study. Once the articles were examined in depth against inclusion and exclusion criteria, discrepancies were discussed to reach a consensus in the final selection of the studies. This first review and discussion of the studies of the 11 articles lead to the elimination of a further six articles that did not adequately fit the inclusion criteria. Thus, a total of 11 articles were finally selected for analysis (Figure 1).

### Quality Assessment

The quality of the selected studies was assessed using a checklist following the methodological guidance for systematic reviews developed by the Joanna Briggs Institute (JBI) (Lockwood et al., 2015). The selected studies were checked against nine questions. The results of the evaluation are presented in the Table 1.

**Table 1:** Quality of studies.

**Source**	**Q1**	**Q2**	**Q3**	**Q4**	**Q5**	**Q6**	**Q7**	**Q8**	**Q9**
Atkins et al., 2015	Yes	Yes	Yes	Yes	Yes	No	Yes	Yes	Yes
Bloemraad and Terriquez, 2016	Yes	Yes	Yes	Yes	Yes	No	Yes	Yes	Yes
Bradshaw et al., 2009	Yes	Yes	Yes	Yes	Yes	No	No	Yes	Yes
Cappella et al., 2012	Yes	Yes	Yes	Yes	Yes	No	Yes	Yes	Yes
Connell and Dishion, 2008	Yes	Yes	Yes	Yes	Yes	No	Yes	Yes	Yes
Fazel, 2015	Yes	Yes	Yes	Yes	Yes	No	Yes	Yes	Yes
Houlston et al., 2011	Yes	Yes	Yes	Yes	Yes	No	Yes	Yes	Yes
Kia-Keating et al., 2017	Yes	Yes	Yes	Yes	Yes	No	Yes	Yes	Yes
McWhirter and McWhirter, 2010	Yes	Yes	Yes	Yes	Yes	No	Yes	Yes	Yes
Ohl et al., 2013	Yes	Yes	Yes	Yes	Yes	No	No	Yes	Yes
Puffer et al., 2016	Yes	Yes	Yes	Yes	Yes	No	Yes	Yes	Yes

Q1. Is there congruity between the stated philosophical perspective and the research methodology?

Q2. Is there congruity between the research methodology and the research question or objectives?

Q3. Is there congruity between the research methodology and the methods used to collect data?

Q4. Is there congruity between the research methodology and the representation and analysis of data?

Q5. Is there congruity between the research methodology and the interpretation of results?

Q6. Is the influence of the researcher on their search, and vice-versa, addressed?

Q7. Are participant, and their voices, adequately represented?

Q8. Is the research ethical according to current criteria or, for recent studies, and is there evidence of ethical approval by an appropriate body?

Q9. Do the conclusions drawn in the research report flow from the analysis or interpretation, of the data?

### Data Analysis

For the analysis of the studies, the three researchers developed an analytical grid to systematize the most relevant information for the purpose of the review: study characteristics, interactions fostered during the intervention, positive effects and information for assessment of the risk of bias. Each researcher analyzed the studies independently aiming at identifying on the one hand, how the interventions promote interactions between different agents, and on the other hand, the effects of the interventions on the target population. Firstly, data was categorized following an inductive method. Secondly, researchers compared their analysis to reach a consensus to report main findings from the review.

## Results

The analysis of 11 mental health interventions targeting children and adolescents reported the benefits for both -students with mental health problems as well as healthy participants- resulting from their participation in the programs analyzed. Nine of the studies show the effects of preventive interventions aim to reduce future problems and to promote mental health among children and adolescents without mental health problems. Only two studies target children who had already contacted the school-based mental health service (Fazel, 2015) and adolescents who presented depressive symptoms (Connell and Dishion, 2008). Overall, the articles reviewed show a series of studies conducted mainly in the US context, seven out of ten, whereas the rest of the studies were carried out in the United Kingdom and Kenya. Seven of the interventions were conducted in schools and four of them were based in the community.

All the studies have shown to promote positive mental health in increasing well-being and preventing other related problems, as well as in reducing affective symptoms among those participants who were already affected. A detailed analysis of the strategies implemented across the mental health programs revealed an emphasis on fostering interactions among the children and adolescents engaging them in dialogues that involved different agents -teachers, families, community members, mental health professionals. An overview of the articles selected is provided in Table 2.

**Table 2:** Summary of included studies.

**First Author - Year**	**Settings**	**Country**	**Assessment instruments**	**Type of study**	**Target population**	Sample size ^*^
Atkins et al., 2015	School	United States	Observations, reports and assessment of academic performance	Intervention/Quasi-experimental/Longitudinal	Children and families	416 (280)
Bloemraad and Terriquez, 2016	Community	United States	Questionnaire, interview, and documentary data	Descriptive/Cross-sectional	Immigrant youth/communities of color	1210 (440)
Bradshaw et al., 2009	School	United States	Academic performance and interview	Intervention/Quasi-experimental/Longitudinal	Children	678
Cappella et al., 2012	School	United States	Questionnaires and interviews	Intervention/Quasi-experimental	Consultant, teacher and children	890 (828)
Connell and Dishion, 2008	School	United States	Questionnaires	Intervention/Longitudinal	At-risk adolescents	998
Fazel, 2015	School	United Kingdom	Interview	Survey/Cross-sectional	Refugee children	40
Houlston et al., 2011	Secondary School	United Kingdom	Questionnaires	Survey/Cross-sectional	Adolescents	334
Kia-Keating et al., 2017	Community	United States	Community forums	Intervention	Latino youth, family, and community	194 (21)
McWhirter and McWhirter, 2010	Community	United States	Group sessions	Comparative intervention	Family and children	
Ohl et al., 2013	School	United Kingdom	Questionnaires	Intervention/Quasi-experimental	Children	385
Puffer et al., 2016	Community	Kenya	Questionnaires	Intervention/Longitudinal	Adolescents	440 (237)

* *Sample size: total sample of the study; brackets indicate total number of children and youth*.

### Supportive Interactions in Mental Health Interventions

Interactions among students, teachers, family, and community members and other professionals play an important role in the interventions analyzed. The mental health programs developed in schools and communities include specific strategies that have an emphasis on enacting peer support, partnerships and dialogic spaces for the children and adolescents to engage in supportive interactions with other relevant peers or adults.

#### Collaborative Interactions Among Children, Teachers and Parents in the School Context

Interactions between teachers and students underpin the strategies of the mental health interventions in different specific ways, which include tutoring, interviews, consultation meetings, peer-assisted learning strategies, interactive games, cooperative non-competitive building games, among others. (Bradshaw et al., 2009; Houlston et al., 2011; Cappella et al., 2012; Ohl et al., 2013; Atkins et al., 2015; Fazel, 2015). Overall, five of the studies implement strategies aim at developing children social skills through interaction and collaboration.

Similarly, interventions focus on “group interactions” as a preventive strategy that seek to reduce future mental health problems and to promote well-being (McWhirter and McWhirter, 2010). Specifically, two group-oriented prevention programs—Project Family Rejuvenation Education and Empowerment and Group-Oriented Psychological Education Prevention- are characterized by small-group discussions among students and with their mothers; in both settings participants engage in dialogue in a nonthreatening climate while encouraging cultivation of feedback and support between them (McWhirter and McWhirter, 2010).

Moreover, three studies promoted collaborative interactions between parents, teachers, and mental health professionals (Bradshaw et al., 2009; McWhirter and McWhirter, 2010; Atkins et al., 2015). Interactive features of these mental health programs include building positive peer groups and partnerships, solving problems peacefully, and fostering parent-student interactions, among others. This aligns with the need for an integration of the school ecology into program planning and the implementation of effective programs, as observed in the Link to Learning (L2L) service model instituted in classrooms and homes to support children with disruptive behavior disorders living in urban low-income communities (Atkins et al., 2015). In the same vein, collaboration between parents and teachers in classrooms is at the heart of the Family-School Partnership Program (Bradshaw et al., 2009). Discussion-based interactions include parents reading aloud to their children, with a particular emphasis in the promotion of reasoning among the students. Interaction is guided-by open-ended questions after the reading or using other materials, such as videotapes. Parents reacted to and discussed the situations and problem-solved alternative approaches. Discussions were also held on problem situations arising at home.

#### Fostering Communicative Skills and Home–School Interaction

Communication skills and family communication practice are a central component of READY—a family-based intervention program to prevent HIV infection and mental health problems (Puffer et al., 2016). The interaction and the communication skills training involved families, caregivers, children, and the community, as the intervention was carried out in religious congregations. By improving family communication as a protective factor against mental health disorders, READY draws on a promising approach to strengthen protective family processes that may prevent future negative outcomes for adolescents (Puffer et al., 2016). In conjunction with these activities, and while the program was being implemented, interaction was also fostered, using a voicemail system to cultivate parents' involvement and to provide consultation on an as-needed basis, and asking parents to fill in and return comment sheets indicating whether they had completed the weekly home activities and whether they had encountered any problems.

For their part, Atkins et al. (2010) carried out an intervention that targeted home-school communication and home routines that support learning, homework support, and daily readings. They promoted interaction between parents and teachers by means of two techniques: Daily Report Cards and Good News Notes. Daily Reports Cards, on the one hand, consist of cards in which teachers and parents join efforts to identify, monitor, and reinforce behaviors that interfere with learning. Teachers and parents agree on a rating system to track behaviors, a reward schedule, and a plan for monitoring intervals that will enhance both direct feedback to students and home-school communication. Good News Notes, on the other hand, are certificates that teachers send to families detailing desirable behaviors exhibited by children, as a means to provide positive weekly feedback to parents. The Notes identify students' strengths, scaffold behavior improvement by reinforcing small achievements, and balance infraction reports with positive feedback.

Overall, these studies report a multilevel approach, tackling schools, families, communities, and mental health providers and services. The three articles include programs that evidence the crucial role of family and parental engagement in promoting mental health among adolescents (Connell and Dishion, 2008; Puffer et al., 2016) and children (Atkins et al., 2015). According to Connell and Dishion (2008), providing family-centered services in the school environment facilitated family engagement in the program.

#### Engaging in Dialogue With Community Members

Engaging in dialogue with the very community members who might be at risk of suffering mental health problems is essential for the success of the intervention. Some strategies for their involvement include the creation of a local Community Advisory Committee (Puffer et al., 2016) or a Community Advisory Board (Kia-Keating et al., 2017). The latter engage participants in reciprocal dialogues on solutions for issues ranging from violence exposure and health disparities to the difficulties encountered by youth people seeking to thrive, as exemplified by the HEROES Project (Kia-Keating et al., 2017).

There have been other community-based organizations studied in California, aimed at promoting “cultures of health” by engaging people in building social networks, by fostering solidarity and collective efficacy, or by promoting a shared commitment to the collective well-being (Puffer et al., 2016). Overall, these programs promote dialogic spaces in which the voices of the minorities, who have often been excluded, are instead given prominence and listened to, in order to look for solutions that will address the inequalities affecting their communities.

### Effects

The effects of the interventions carried out in schools and communities with an emphasis on fostering supportive interactions as discussed above have benefited children and adolescents as reported in the following dimensions:

Internalizing symptomatology: Three studies include interventions that have had positive effects on the treatment and prevention of affective disorders such as depression and anxiety. Thus, Connell and Dishion (2008) ascertained, throughout 3 years, their potential to reduce and prevent the increase of depressive symptoms in at-risk early adolescents. Likewise, Ohl et al. (2013) confirmed the effectiveness of relationships for decreasing emotional symptoms. McWhirter and McWhirter (2010) garnered the results of the GOPEP intervention program (McWhirter et al., 1997), based on group sessions and on conjoint sessions, which entailed substantial collaboration between researchers and participants, and confirmed the effectiveness of the SOAR program (Student Optimistic Attitudes and Resilience Program) in reducing anxiety and depression. The FREE program, for its part, was effective in decreasing self-isolation among children and their mothers, survivors of domestic violence.
Externalizing symptomatology: Four articles present improvements in aspects related to aggression and behavioral issues. Ohl et al. (2013) confirmed that the Pyramid project helped improve peer problems; however, they did not find positive effects on behavioral problems. McWhirter and McWhirter (2010) gathered evidence confirming the effectiveness of the FREE project in decreasing family conflict, and of the SCARE (Student-Created Aggression Replacement Education) program, one of the GOPEP intervention programs, in decreasing and managing aggression, anger, and violent behaviors. However, Cappella et al. (2012) did not find significative differences in behavioral regulation as an effect of their BRIDGE intervention, although children identified as having behavioral problems benefitted more than their peers in the area of social relations. On the other hand, Bradshaw et al. (2009) confirmed the long-term positive effects in reducing behavioral and mental-health problems resulting of the CC intervention.
Personal well-being: Six of the studies reported positive effects on strengthening psychological-related aspects to well-being, including self-concept, self-esteem, self-efficacy, and empowerment, among others. Cappella et al. (2012) confirmed the existence of a positive effect of intervention on children's academic self-concept. Atkins et al. (2015) found a significantly greater improvement on social skills among children who had been involved in the intervention, whereas Ohl et al. (2013) ascertained its positive effect on prosocial behavior. Houlston et al. (2011) confirmed that peer support improves self-esteem in victims of bullying, as well as their perception of the support provided by friends and other students. Participants stated that peer support had a positive impact on students' relationships, improving and building peer networks with trained peer supporters and other students. More specifically, in bullying situations, students considered peer support to be helpful for a number of reasons, including being able to talk about it, having peers to interact with, or helping bullied students to tell others of their plight.
Bloemraad and Terriquez (2016) gathered the opinions of people taking part in activities organized by CBOs (Community-Based Organizations). Results provide evidence of the impact that involvement in CBOs has on participants, namely when preparing to enroll and succeed in college, as well as on their self-reported civic capacity developing skills, which encompass skills as diverse as communicating with others, understanding the impact that government decisions have on the community, speaking in public, or planning events. Besides, the involvement in CBOs improves personal empowerment and self-efficacy, as participants learn to stand up for their beliefs, become more aware of health issues impacting their communities, and learn about their own culture or ethnic group. As for health and education outcomes, participants became more informed about college and career options, took better care of their personal health, and improved their school grades.
McWhirter and McWhirter (2010) showed that the FREE project resulted in an increase in children's and women's emotional well-being, peer engagement and self-esteem in children, as well as women's self-efficacy.
Context: Five of the interventions reported improvements on the classroom climate and teacher-student and peer interactions. The study carried out by Cappella et al. (2012), based on BRIDGE intervention, demonstrates how classroom interactions generate a positive climate where emotional support and teacher sensitivity are prominent. These interactions also promote a positive classroom climate, characterized by optimal behavior management, productivity, and instructional learning formats. Furthermore, they have been verified to help develop instructional support, more positive teacher expectations regarding children's behavior, and a more responsive teacher-student relationship. The study by McWhirter and McWhirter (2010), based on group interventions, highlights that interacting with other people helps build positive peer/adult relationships. These conclusions are shared by Puffer et al. (2016), whose study focused on family communication, and who conclude that intra-family communication improves well-being. In a similar vein, Bloemraad and Terriquez (2016) find that the interactions fostered by the intervention improve well-being in the community.

## Discussion

The present systematic review of studies has fulfilled the objective of identifying evidence for positive effects of interaction-based interventions in schools and communities in children's and adolescents' mental health. We have shown that mental health interventions, in which supportive interactions are fostered among different actors, have a positive effect in decreasing affective symptoms and in increasing personal wellbeing among children and adolescents.

We detected in these programs an emphasis on engaging children and adolescents in supportive interactions with other relevant adults, such as teachers, family, community members, and other professionals. Overall it showcases the benefits children and adolescents without mental health problems can reap, particularly in preventive interventions as nine of the studies focused on. Only two studies target adolescents and children with mental health problems (Connell and Dishion, 2008; Fazel, 2015). The literature analyzed sheds light on the importance of preventive interventions where different agents work together toward the common goal of promoting children's and adolescents' mental health (Atkins et al., 2015; Kia-Keating et al., 2017).

Positive effects on mental health are achieved through interventions that are culturally appropriate and culturally grounded (Bloemraad and Terriquez, 2016; Puffer et al., 2016; Kia-Keating et al., 2017). This is particularly important in those interventions which require the active engagement of families and community members. The role of family and community members emerges as particularly relevant and providing them with communicative skills and fostering home-school communication are assets for the mental health interventions. Schools thus become an ideal space to facilitate family and community involvement, and they consequently present a great potential for enhancing positive parent-teacher, teacher-student and student-student interactions. This is consistent with other research that has focused on the benefits of school-based mental health interventions to help them develop cognitively, socially, and emotionally (Fazel et al., 2014).

There is enough supporting evidence on the potential of these interventions for schools to create a positive climate based on instructional and emotional support, solidarity and friendship that improves the well-being of children and communities (McWhirter and McWhirter, 2010; Bloemraad and Terriquez, 2016; Puffer et al., 2016). Available evidence on the effectiveness of these studies attests to the attainment of positive gains in students' academic achievement, which will also lead to other long-term positive effects that will help prevent behavioral and mental-health problems (Bradshaw et al., 2009). This positive effect is particularly strong in high poverty contexts (Atkins et al., 2015). Particularly relevant is the reduction of anxiety and depression, especially in light of the marked increase of the latter, currently ailing 4,4% of the world population (WHO, 2017).

Overall, we argue that interaction-based approaches in mental health interventions, that involve diverse actors in productive forms of dialogue and supportive interactions, are consistent with the benefits reported by the sociocultural approaches to learning and development (Vygotsky, 1978). However, in this systematic review we have not been able to determine the effect of the interaction on the effectiveness of the intervention. This is consistent with the literature, as effective mental health interventions, which include collective interactions among different agents as a central element of the intervention, did not revealed how those interactions were linked to the positive outcomes obtained (Seikkula and Arnkil, 2006). Similarly, the primary studies reviewed do not established a direct link of the interaction component of the intervention with the positive mental health outcomes. This question still remains.

### Limitation and Future Directions

In this systematic review we have reviewed a set of interventions for both adolescents and children, without explicitly distinguishing within the two study groups. This raises a limitation as children and adolescents can potentially show different needs in terms of mental and behavioral support. Consequently, there could be potential differences in the outcomes that have not been considered in this review. In the same vein, this study only reviewed research in English and most research was conducted in the United States, which could also limit the generalizability of the results.

On the other hand, the concept of interaction we explored it is a broad concept that presents some limitations in providing a consistent definition within the interventions. Furthermore, the primary studies reported the effects of the intervention as a whole. Therefore, their methodological designs do not allow to identify the specific effect on mental health of the interaction itself. Still there is a gap to determine the effect of the interactions on the mental health outcomes. Further research is needed to explore the particular role and potential of social interaction to promote children and adolescents' mental health.

## Conclusions

This systematic review of 11 studies has focused on mental health interventions in which interaction plays an important role. Supportive interactions carried out in the framework of mental health interventions involve various contexts, agents and systems, including teachers, parents, mental-health professionals, and members of the community.

There is evidence of a positive effect on the mental health of children and adolescents, both in decreasing internalizing and externalizing symptoms, and in promoting personal well-being. Factors that foster mental health as social support or engagement also increase with interventions programs that include interaction as a main feature.

However, more research is needed into the specific impact of interaction on the mental health of children and adolescents, as well as analyzing the type of interactions that have the most beneficial effect.

## Author Contributions

RG-C wrote the proposal of this systematic review with the input and contributions of the research team BV-C and LV-G. RG-C and LV-G planned the search in databases and defined exclusion and inclusion criteria for the selection of the articles. BV-C carried out the search, screen the materials and proposed a selection. All the authors checked and refined the selection of the studies. Each author drafted a section of this manuscript. All authors reviewed the whole manuscript, read and approved the submitted version.

### Conflict of Interest Statement

The authors declare that the research was conducted in the absence of any commercial or financial relationships that could be construed as a potential conflict of interest.
